# Biatrial volume ratio predicts low voltage areas in atrial fibrillation

**DOI:** 10.1002/clc.23720

**Published:** 2021-09-08

**Authors:** Timm Seewöster, Borislav Dinov, Sotirios Nedios, Gerhard Hindricks, Philipp Sommer, Jelena Kornej

**Affiliations:** ^1^ Department of Electrophysiology Heart Center Leipzig – University Hospital of Cardiology Leipzig Germany; ^2^ Department of Electrophysiology Herz‐ und Diabeteszentrum NRW Bad Oeynhausen Germany; ^3^ School of Medicine – Cardiovascular Medicine Boston University Boston Massachusetts USA

**Keywords:** atrial fibrillation, cardiac magnetic resonance, left atrial size, low voltage areas, right atrial volume

## Abstract

**Background:**

Left atrial volume (LAV) and low voltage areas (LVAs) are acknowledged markers for worse rhythm outcome after ablation of atrial fibrillation (AF). Some studies reported the importance of increased right atrial volume (RAV) as a predictor for arrhythmia recurrences in AF patients.

**Objective:**

To investigate association between the LAV/RAV ratio and LVAs presence.

**Methods:**

Patients undergoing first AF ablation were included. LVAs were assessed peri‐procedurally using high‐density 3D maps and defined as <0.5 mV. All patients underwent pre‐procedural cardiovascular magnetic resonance imaging. LAV (biplane) and RAV (monoplane 4‐chamber) were assessed prior to ablation, and the LAV/RAV ratio was calculated.

**Results:**

The study population included 189 patients (age mean 63 ± 10 years, 33% women, 57% persistent AF, 22% LVAs). There were 149 (79%) patients with LAV > RAV. In univariable analysis LAV > RAV was associated with LVAs (OR 6.803, 95%CI 1.395–26.514, *p* = .016). The association remained robust in multivariable model after adjustment for persistent AF, CHA_2_DS_2_‐VASc score, and heart rate (OR 5.981, 95%CI 1.256–28.484, *p* = .025). Using receiver operator curve analysis, LAV > RAV (AUC 0.668, 95%CI 0.585–0.751, *p* = .001) was significant predictor for LVAs. In multivariable analysis, after adjustment for age, persistent AF, and renal function, RAV≥LAV was threefold higher in males (OR 3.040, 95%CI 1.050–8.802, *p* = .04).

**Conclusions:**

LAV > RAV is useful for the prediction of electro‐anatomical substrate in AF. LAV > RAV was associated with LVAs presence, while male sex remained associated with RAV≥LAV and less LVAs.

## 1 INTRODUCTION

Becoming a cornerstone therapy in many patients with atrial fibrillation (AF),^1^ in some patients, catheter ablation with circumferential pulmonary vein isolation alone is not enough for sinus rhythm maintenance during follow‐up. The arrhythmia recurrences remain an important clinical challenge and require individualized AF treatment plan already before intervention. One major feature reflecting left atrial (LA) remodeling is peri‐procedural evidence of low voltage areas (LVAs).^2^ At least 20%–25% of AF patients have significant LVAs in peri‐procedural mapping, which are an important characteristic of AF progression and treatment failure if not treated with additional ablation.^2^, ^3^ Therefore, assessment of LVAs presence before catheter ablation is an important task for the electrophysiologist allowing individually tailored AF ablation therapy.

The LA size is another important parameter of AF progression. The role of the LA volume (LAV) and LA function as surrogate parameters for higher AF burden^4^ and LVAs presence^5^, ^6^ are well described. In addition, there is an association between atrial flutter—a right atrial (RA), and AF—a left atrial disease.^7^, ^8^ Several studies described an importance of RA assessment as a prognosis marker in heart failure, pulmonary hypertension, and chronic obstructive pulmonary disease.^9^, ^10^, ^11^ Diastolic functional changes—as a preliminary stage for AF—appeared to occur earlier in the right chambers^12^ suggesting that RA dilatation might be an early marker for atrial remodeling associated with AF initiation. Previous studies reported the importance of increased RA volume (RAV) as predictor for arrhythmia recurrences in AF patients.^13^, ^14^ It was hypothesized that RA is more prone to hemodynamic changes and is a more responsive marker of structural remodeling.^13^ However, association between RAV and LVAs and the prediction capability for LAV/RAV ratio is unknown. Therefore, we aimed to investigate the indexed LAV/RAV ratio assessed in cardiovascular magnetic resonance (CMR) imaging and the association with LVAs in patients undergoing AF catheter ablation. We hypothesize that the biatrial ratio is an independent predictor for LVAs presence.

## 2 METHODS

The study population was described previously.^6^ Briefly, patients presenting for catheter ablation due to symptomatic AF from October 2015 to April 2017 were included in the study. According to current guidelines, AF subtypes were defined as paroxysmal and persistent.^15^ Patients with pregnancy, age <18 or >75 years, valvular AF (any valvulopathies >second degree), cancer, acute, or systemic inflammatory diseases, and acute hyperthyreotic state were excluded from the study. The study was approved by the local Ethical Committee (Medical Faculty, University of Leipzig), and patients provided written informed consent for participation.

### 2.1 Cardiovascular magnetic resonance

Prior to AF catheter ablation, all patients underwent 1.5 T CMR (Ingenia, Philips Medical) for LA anatomy assessment as previously described.^5^ Briefly, LAV was determined using a biplane model based on cine 4‐ and 2‐chamber views, and RAV using a monoplane model based on the cine 4‐chamber view. Both volumes were indexed to body surface areas, and the LAV/RAV ratio was calculated before ablation. We defined two subgroups according to the LAV/RAV ratio: (1) LAV is greater than RAV (LAV > RAV), and (2) RAV is equal or greater than LAV (RAV ≥ LAV).

### 2.2 Peri‐procedural LA mapping and AF ablation

Transseptal access and catheter navigation were performed with a steerable sheath (Agilis, St. Jude Medical, St. Paul, MN). The electro‐anatomical mapping was performed in sinus rhythm as described previously.^5^ In case of AF at the beginning of the procedure, the arrhythmia was terminated by electrical cardioversion and the mapping was performed in sinus rhythm.

Multielectrode spiral mapping catheters (Reflexion Spiral and Advisor, St Jude Medical [SJM], Saint Paul, MN in NavX Ensite procedures and Carto Lasso, Biosense Webster, Diamond Bar, CA in Carto3 procedures) were used to generate electro‐anatomical voltage maps of the LA. The cutoff value for LVAs was defined as bipolar signal amplitude <0.5 mV. Ectopic beats were excluded from the voltage map. The number of points obtained was >1000. In case of AF recurrence during electro‐anatomical mapping, only anatomical map was assessed. Then the electro‐anatomical mapping was completed in sinus rhythm after PVI, and if still needed, after further cardioversion.

All patients received circumferential ablation lines around the antrum of the ipsilateral pulmonary veins. The ablation catheters (irrigated tip catheter and power of 25–40 W) used in NavX Ensite procedures were TactiCath (St Jude Medical [SJM], Saint Paul, MN) and for Carto3 procedures SmartTouch Thermocool (Biosense Webster, Diamond Bar, CA). End point of catheter ablation was PV isolation, which was verified with a multipolar circular mapping catheter. Additional linear lesions were added between if LVAs were present in this area. To be considered as relevant the LVAs ought to consist of adjacent low‐amplitude mapping points, bearing certain additional characteristics such as fragmentation and duration. Finally, the relevance of the LVAs was evaluated by experienced operators based on the mapping point qualities as well as induction of extra‐PV macro‐reentry tachycardia, in which case additional linear ablation was performed. Patients with small/negligible LVAs dispersely distributed in LA and not suitable for ablation, who received the PVI only without additional ablation lines, were excluded.

### 2.3 Statistical analysis

Data are presented as mean and standard deviation for normally distributed or median (interquartile range, 25th and 75th percentiles) for skewed continuous variables, and as proportions for categorical variables. The differences between continuous values were assessed using an unpaired *t*‐test or the Mann–Whitney, and a *χ* ^2^ test for categorical variables.

Logistic regression analysis was used to identify factors associated with LVAs. We performed three analyses using logistic regression of LVAs presence (**Model 1** – unadjusted analysis; **Model 2 –** adjusted for age and sex; and **Model 3** – adjusted for persistent AF, heart rate, and CHA_2_DS_2_‐VASc score.

Receiver operating characteristic curves (ROC) were generated to analyze performance of the LAV/RAV ratio predicting LVAs, with the area under the curve (AUC) being equivalent to the c‐index for determining the predictive value for the parameters. Finally, we compared the c‐indices (i.e., areas under the ROC curves) of LAV > RAV and LAV using DeLong's method.^16^ A *p*‐value <.05 was considered statistically significant. All analyses were performed with SPSS statistical software version 26 (SPSS Inc., Chicago).

## 3 RESULTS

### 3.1 Clinical characteristics of the study population

The study population included 189 patients (mean 63 ± 10 years, 33% women, 57% persistent AF) undergoing their first AF catheter ablation. Clinical characteristics of the study population are summarized in Table 1. Forty‐one patients (22%) had LVAs in periprocedural mapping, which required additional substrate modification. We observed LAV > RAV in 149 (79%) patients (26% with LVAs), while 40 (21%) patients had RAV≥LAV (5% with LVAs). Patients with LAV > RAV were significantly older, more often females, had a lower estimated glomerular filtration rate, had more often hypertension and more often LVAs (Table 1).

**TABLE 1:** Clinical characteristics of the study cohort

	Total study population	LAV > RAV		*p*‐value
	*n* = 189	Yes (*n* = 149)	No (*n* = 40)	
Age, years	63 ± 10	64 ± 9	58 ± 13	.005
Females, %	33	39	13	.002
Persistent AF, %	57	60	45	.095
LVAs, %	22	26	5	.004
BMI, kg/m^2^	29.7 ± 5.5	29.5 ± 4.7	30.4 ± 7.7	.349
eGFR, ml/min/1.73m^2^	78 ± 18	75 ± 17	86 ± 17	.001
Hypertension	79	82	67	.036
Diabetes mellitus	22	23	19	.648
Chronic heart failure	10	9	14	.355
CHA_2_DS_2_‐VASc score	2.5 ± 1.6	2.6 ± 1.6	2.2 ± 1.7	.142
RA volume, ml/BSA	44 ± 18	40 ± 13	59 ± 27	<.001
LA volume, ml/BSA	59 ± 18	62 ± 18	48 ± 17	<.001
Heart rate, bpm	79 ± 24	81 ± 25	74 ± 30	.069

Abbreviations: AF, atrial fibrillation; BMI, body mass index; BSA, body surface area; eGFR, estimated glomerular filtration rate; LA, left atrial; LVAs, low voltage areas; RA, right atrial.

### 3.2 Association of LAV/RAV ratio with LVAs

**FIGURE 1:**
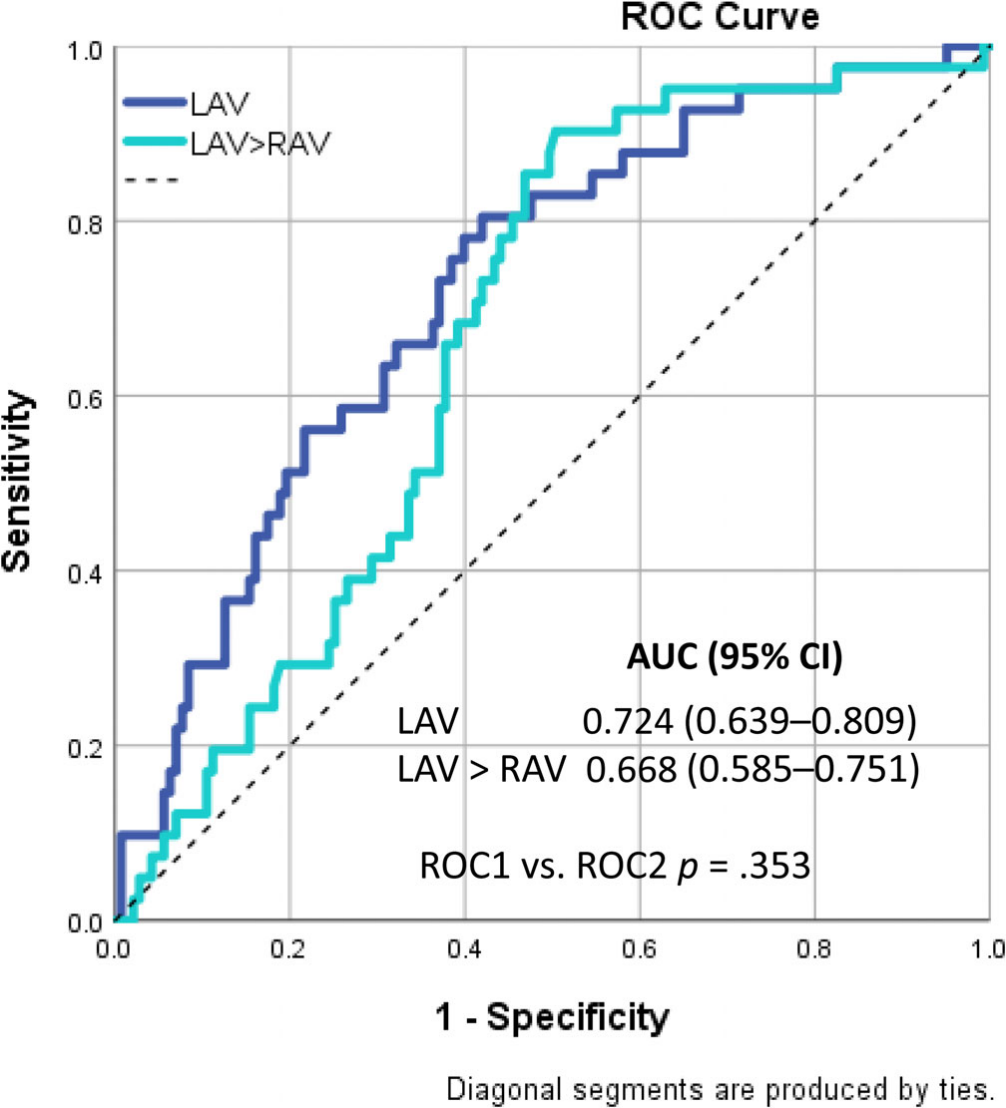
Association between LVAs and LAV > RAV. AF, atrial fibrillation; AUC, area under the curve; CI, confidence interval; LAV, left atrial volume; RAV, right atrial volume

In univariable analysis logistic regression, LAV > RAV was associated with sixxfold risk of LVAs presence (OR 6.083, 95%CI 1.395–26.514, *p* = .016). In multivariable analysis, after adjustment for CHA_2_DS_2_‐VASc score, persistent AF, and heart rate, LAV > RAV remained significantly associated with LVAs (OR 5.981, 95%CI 1.256–28.484, *p* = .025) (Table 2). Using ROC analysis, LAV > RAV showed moderate prediction of LVAs presence (AUC 0.668, 95%CI 0.585–0.751, *p* = .001) (Figure 1). Comparing the LAV > RAV with LAV (AUC 0.724, 95% CI 0.639–0.809, *p* < .001) using DeLong's method, the difference between ROC curves was not significant (*p* = .353).

**TABLE 2:** Prediction of LVAs using LAV/RAV and LA volume

	Unadjusted model		Adjusted for age and sex		Multivariable model^a^	
	OR (95% CI)	*p*‐value	OR (95% CI)	*p*‐value	OR (95% CI)	*p*‐value
LAV > RAV	6.083 (1.395–26.514)	.016	4.257 (0.933–19.432)	.061	5.981 (1.256–28.484)	.025
LA volume (indexed)	1.042 (1.021–1.063)	<.001	1.039 (1.018–1.061)	<.001	1.031 (1.009–1.054)	.007

Abbreviations: AF, atrial fibrillation; CI, confidence interval; LAV, left atrial volume; OR, odds ratio; RAV, right atrial volume.

^a^ Further adjusted for persistent AF; CHA_2_DS_2_‐VASc score and heart frequency.

### 3.3 Clinical factors associated with RAV ≥ LAV

In univariable analysis, RAV ≥ LAV was associated with younger age (OR 0.946, 95% CI 0.914–0.978, *p* = .001), male sex (OR 4.462, 95% CI 1.652–12.046, *p* = .003), and renal function measured as glomerular filtration rate (OR per 1 ml/kg/1.73m^2^ 1.041, 95% CI 1.016–1.066, *p* = .001, Table 3). In multivariable analysis, the RAV ≥ LAV was 3‐fold higher in males (OR 3.040, 95% CI 1.050–8.802, *p* = .04).

**TABLE 3:** Clinical factors associated with RAV ≥ LAV

	Univariable analysis		Multivariable analysis^a^	
	OR (95% CI)	*p*‐value	OR (95% CI)	*p*‐value
Age	0.946 (0.914–0.978)	.001	0.959 (0.919–1.002)	.061
Persistent AF	0.552 (0.273–1.115)	.098	0.531 (0.237–1.188)	.123
Males	4.462 (1.652–12.046)	.003	3.040 (1.050–8.802)	.040
eGFR	1.041 (1.016–1.066)	.001	1.018 (0.988–1.050)	.243
CHA_2_DS_2_‐VASc score	0.844 (0.673–1.059)	.143		

Abbreviations: AF, fibrillation; CI, confidence interval; eGFR, estimated glomerular filtration rate; OR, odds ratio.

^a^ Adjusted for age, sex, persistent AF, and eGFR.

## 4 DISCUSSION

**FIGURE 2:**
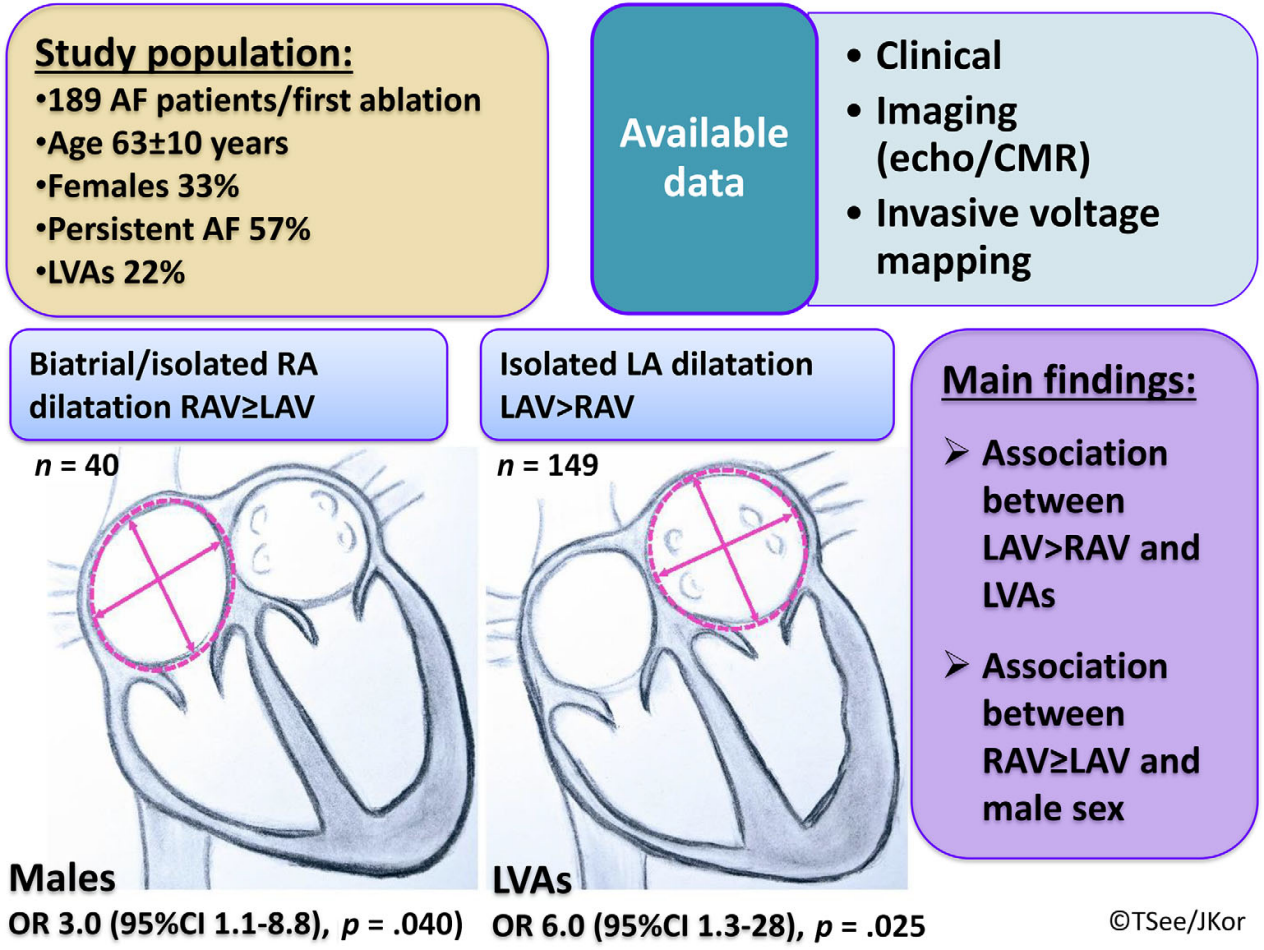
Study overview. Central figure describes the study population and main results. Isolated LA dilatation (LAV > RAV) was significantly associated with LVAs, while biatrial/isolated RA dilatation (RAV≥LAV) was associated with male sex. AF, atrial fibrillation; CI, confidence interval; CMR, cardiovascular magnetic resonance imaging; HR, heart rate; LA, left atrial; LAV, left atrial volume; LVAs, low voltage areas; RA, right atrial; RAV, right atrial volume; OR, odds ratio

In our study, we investigated the indexed LAV/RAV ratio and its predictive value on the pre‐procedural LVAs in patients undergoing AF catheter ablation (Figure 2). We found that LAV > RAV was associated with sixfold risk for LVAs presence. Also, LAV > RAV was less observed in males.

### 4.1 Biatrial ratio as parameter for AF progression and LVAs prediction

The role of RA size in AF pathogenesis is controversial. It had been reported that RAV and the RAV/LAV ratio were predictive for AF recurrence after PVI in patients with persistent AF, while LAV was not.^14^ Another study confirmed these findings in AF patients after cardioversion.^13^ Both studies suggested that AF is a biatrial disease, not being exclusively associated with isolated LA remodeling. However, other studies reported only moderate^17^ or weak^18^ prediction of AF recurrences using echocardiographic RA diameter. Our study contradicts previous results and shows that RAV ≥ LAV was associated with less LVAs presence, suggesting a rather minor role in LA remodeling. However, we found that males had threefold higher odds for RAV ≥ LAV and less LVAs. Taking into account that females are more prone to LVAs compared to male^19^ and have more often‐unfavorable outcomes after catheter ablation, our findings are in line with previous research calling for an attention and an urgent need to address underrepresentation of females in clinical research.

### 4.2 Clinical implications

LA diameter (LAD) is an acknowledged marker of advanced electro‐anatomical remodeling.^2^, ^20^, ^21^ LA remodeling is associated with increased atrial volume, interstitial fibrosis, and increased myocardial stretch favoring AF sustainability.^22^ Previously, we reported that besides anteroposterior LAD, the LAV assessed in CMR is a strong predictor for LVAs presence.^6^ In current analysis we confirm the role of LA in AF pathogenesis showing that LAV > RAV was associated with sixfold risk for LVAs presence. Although LAV alone showed better predictive value than LAV > RAV (AUC 0.724 vs. 0.668), the difference between ROC curves was not significant. However, the risk of LVAs presence was more obvious using LAV/RAV ratio than LAV alone (OR 5.98 vs. 1.03). Our results indicate that the LA enlargement indexed for the RAV (as self‐reference for enlargement) as reflected with the LAV/RAV ratio is a helpful tool for LVAs prediction and for shaping an individualized AF management approach prior to AF catheter ablation.

The present findings add to our knowledge about the importance of side‐specific atrial remodeling. As previously described, LA remodeling is associated with later stages of AF progression resulting from risk factors like aging, hypertension, left ventricular (LV) diastolic dysfunction and an altered electromechanical activation.^23^, ^24^, ^25^, ^26^ LV stiffness results into higher LA pressure with reduced LA emptying and consequent atrial dilatation.^23^ Described pathologic changes represent a common pathway associated with interatrial delay seen as biphasic P‐wave in ECG and caused by deterioration of the Bachmann bundle conduction, and finally impaired electromechanical LA activation.^25^ These pathophysiologic changes contribute to advanced remodeling and wall deformation that has been associated with LVA.^27^ Our study supplement these findings of side specific pathophysiological LA changes (especially in relation to RAV) and emphasize the need for accurate pre‐procedural LA assessment.

In contrast, pathophysiology of RA remodeling seems to be different. In patients without heart failure, volume and pressure overload in the RA is mainly associated with pulmonary resistance, valvular disease, and RV dysfunction.^28^, ^29^, ^30^ Although AF may contribute to RA dilation as well,^31^ AF triggers from the RA are rare^32^ and RA ablation in AF patients has not shown any benefit for outcomes.^33^ In our study, RAV > LAV was higher in males and was not associated with LVAs. An explanation for such sex‐specific difference remains unknown, but it is in accordance with large echocardiographic studies and has not been assigned any clinical significance.^34^, ^35^, ^36^

### 4.3 Future directions

Our findings show a strong association between LVAs and LAV/RAV ratio. Despite previous data reporting a correlation between RAV ≥ LAV and AF recurrences, our results imply that increased RAV is not a suitable parameter for LVAs prediction and therefore not a marker for left atrial myopathy. This is in line with our previous studies that emphasized the importance of LV diastolic dysfunction, electro‐anatomical dysfunction for asymmetric LA remodeling, and ablation outcomes.^23^, ^24^, ^25^ Future studies should thus focus on the LA size and its proportional enlargement in relation to the RA, when assessing patients prior to an AF ablation procedure.

### 4.4 Limitations

Several limitations could impact interpretation of our results. First, the study is relatively small and included only 33% women. Therefore, statistical analysis for females is very likely underpowered. Similarly, we cannot exclude that male sex is a possible effect modifier analyzing an impact of sex for RAV ≥ LAV. Of note, the study included only patients of European ancestry from a localized area in Eastern Germany, limiting transferability of the results to other ethnic populations. Furthermore, although LVAs are one of parameters describing atrial myopathy, other parameters such as extrapulmonary substrate and triggers or typical atrial flutter, impairing AF ablation outcome, were not assessed. We did not consider arrhythmia recurrences as an outcome after AF catheter ablation due to several issues: (1) In our opinion LVAs are more robust outcome than arrhythmia recurrences, which occur within weeks/months after catheter ablation, while LVAs are measurable during the procedure; (2) Arrhythmia recurrences depend on many factors such as ablator experience, follow‐up strategy including antiarrhythmic drug prescription, follow‐up frequency, ECG monitoring; (3) In our study, patients did not have a continuous rhythm monitoring. Finally, specific LA fibrosis assessment using late gadolinium enhancement was not conducted in current study and should be addressed in future studies.

## 5 CONCLUSIONS

LAV/RAV ratio is useful parameter predicting electro‐anatomical substrate in AF. LAV > RAV was associated with sixfold risk for LVAs presence, while male sex was associated with RAV ≥ LAV and less LVAs.

## CONFLICT OF INTEREST

Philipp Sommer is in the advisory board for Abbott, Biosense Webster, Medtronic, und Boston Scientific.

## Data Availability

Data sharing is not applicable to this article as no new data were created or analyzed in this study.
